# Beyond Support Cells: Astrocytic Autophagy as a Central Regulator of CNS Homeostasis and Neurodegenerative Diseases

**DOI:** 10.3390/cells14171342

**Published:** 2025-08-29

**Authors:** Jung Ho Lee, Wonseok Chang, Sun Seek Min, Dae Yong Song, Hong Il Yoo

**Affiliations:** 1 Department of Pharmacology, Eulji University School of Medicine, Daejeon 34824, Republic of Korea; jungholee@eulji.ac.kr; 2 Department of Physiology and Biophysics, Eulji University School of Medicine, Daejeon 34824, Republic of Korea; cws2017@eulji.ac.kr (W.C.); ssmin@eulji.ac.kr (S.S.M.); 3 Department of Anatomy and Neurosciences, Eulji University School of Medicine, Daejeon 34824, Republic of Korea; dysong@eulji.ac.kr; 4 Department of Biomedical Engineering, Johns Hopkin University School of Medicine, Baltimore, MD 21205, USA

**Keywords:** astrocyte, autophagy, neurodegenerative disease, therapeutics

## Abstract

Autophagy is a fundamental catabolic pathway critical for maintaining cellular homeostasis in the central nervous system (CNS). While neuronal autophagy has been extensively studied, growing evidence highlights the crucial roles of astrocytic autophagy in CNS physiology and pathology. Astrocytes regulate metabolic support, redox balance, and neuroinflammatory responses. These functions are closely linked to autophagic activity. The disruption of astrocytic autophagy contributes to synaptic dysfunction, chronic inflammation, myelin impairment, and blood–brain barrier instability. Dysregulation of astrocytic autophagy has been implicated in the pathogenesis of multiple neurodegenerative diseases, including Alzheimer’s disease, Parkinson’s disease, Huntington’s disease, and amyotrophic lateral sclerosis. This review summarizes the molecular mechanisms of autophagy in astrocytes and delineates its role in intercellular communication with neurons, microglia, oligodendrocytes, and endothelial cells. Furthermore, we will discuss current pharmacological approaches targeting astrocytic autophagy, with particular attention to repurposed agents such as rapamycin, lithium, and caloric restriction mimetics. Although promising in preclinical models, therapeutic translation is challenged by the complexity of autophagy’s dual roles and cell-type specificity. A deeper understanding of astrocytic autophagy and its crosstalk with other CNS cell types may facilitate the development of targeted interventions for neurodegenerative diseases.

## 1. Introduction

Autophagy is an evolutionarily conserved cellular mechanism that serves as a major degradation and recycling pathway in eukaryotic cells [1]. There are three primary types of autophagy: macroautophagy, microautophagy, and chaperone-mediated autophagy [2]. By eliminating damaged organelles, aberrant proteins, and other cellular debris, autophagy plays a pivotal role in maintaining cellular homeostasis and is essential for development, immune regulation, and disease prevention [3].

Within the central nervous system (CNS), autophagy performs several critical functions. In particular, autophagy in astrocytes—the most abundant glial cells in the CNS—plays a multifaceted and indispensable role in maintaining CNS homeostasis [4]. Beyond its well-established functions, astrocytic autophagy is increasingly recognized as a key modulator of neuronal activity through interactions with various brain cell types, including endothelial cells, microglia, and oligodendrocytes.

Neurological diseases impose a substantial global health burden, affecting millions of individuals and leading to severe disability and reduced quality of life [5]. Disorders such as stroke, Alzheimer’s disease (AD), Parkinson’s disease (PD), Huntington’s disease (HD), and amyotrophic lateral sclerosis (ALS) are characterized by progressive neuronal dysfunction and loss, highlighting the urgent need for innovative therapeutic strategies. While traditional approaches have primarily focused on neurons, growing evidence has advanced our understanding of the critical role of glial cells—particularly astrocytes—in the pathophysiology and progression of these diseases.

When autophagy in astrocytes is impaired, the maintenance of the brain microenvironment may be disrupted, potentially resulting in functional deficits and uncontrolled neuroinflammation [6]. Furthermore, given the autophagic alterations observed in various neurodegenerative diseases, it is plausible that astrocytic autophagy plays a central role under these pathological conditions. Thus, pharmacological modulation of autophagy may help restore cellular homeostasis, facilitate the clearance of disease-related proteins, and ultimately mitigate the symptoms and progression of neurological disorders.

Although our understanding of these mechanisms has advanced considerably, the specific pharmacological targeting of astrocytic autophagy remains a significant challenge [7]. In the following sections, we review current experimental findings and evidence that underscore the importance of astrocytic autophagy. Furthermore, given that drug development specifically targeting astrocytic autophagy represents an emerging area of drug development, we will also examine the status of CNS-directed autophagy-modulating drugs and assess the experimental evidence related to the regulation of autophagy in astrocytes.

## 2. Molecular Insights of Autophagy in the CNS

### 2.1. Molecular Mechanisms of Autophagy

Autophagy is a fundamental catabolic process that maintains cellular homeostasis through the degradation of cytoplasmic materials via the lysosomal pathway [8,9]. Among the three major types of autophagy—macroautophagy, chaperone-mediated autophagy (CMA) [10], and microautophagy [11]—macroautophagy is the most widely studied and often referred to simply as ‘autophagy’. This highly regulated and dynamic process is crucial for proteostasis, organelle quality control, and cellular adaptation to stress [12].

#### Macroautophagy

Macroautophagy is characterized by the de novo formation of a double-membrane vesicle known as the autophagosome, which engulfs portions of the cytoplasm, including damaged organelles, protein aggregates, or invading pathogens. The process is initiated in response to various cellular signals, particularly energy depletion or nutrient deprivation. Under such conditions, the mechanistic target of rapamycin complex 1 (mTORC1), a major negative regulator of autophagy, becomes inactivated [13,14], while AMP-activated protein kinase (AMPK) becomes activated [15]. These shifts converge on the activation of the ULK1/2–FIP200–ATG13 complex [16], which serves as the primary regulatory node for autophagy initiation.

Following initiation, a nucleation complex composed of Beclin-1, VPS34 (Class III PI3K), ATG14L, and VPS15 orchestrates the generation of phosphatidylinositol 3-phosphate (PI3P), facilitating the recruitment of downstream effector proteins to the membrane source—often derived from the endoplasmic reticulum (ER)—to begin the formation of the phagophore, the precursor to the autophagosome [17].

The elongation and maturation of the phagophore involve two ubiquitin-like conjugation systems: the ATG12–ATG5–ATG16L1 complex and the LC3 (microtubule-associated protein 1 light chain 3) lipidation pathway. The ATG12–ATG5–ATG16L1 complex is assembled and localized to the expanding phagophore [18,19]. In parallel, LC3 is proteolytically cleaved to LC3-I and then lipidated to form LC3-II [20], which is associated with autophagosome membranes. LC3-II serves not only as a structural component but also as a critical docking site for cargo adaptor proteins such as p62/SQSTM1, NDP52, and NBR1, which link specific cargo to the forming autophagosome through LC3-interacting regions (LIRs) [21,22,23].

Once fully formed, the autophagosome is trafficked along the cytoskeleton—primarily via dynein-mediated retrograde transport—to fuse with lysosomes [24]. This fusion is facilitated by SNARE proteins, Rab7 GTPases, and the endosomal sorting complex required for transport (ESCRT) machinery [25]. The resultant autolysosome contains lysosomal hydrolases that degrade the sequestered material into basic metabolites, such as amino acids and fatty acids, which are then recycled back into the cytosol to support anabolic pathways or cellular energy demands.

Beyond non-selective degradation, macroautophagy has evolved to encompass highly selective forms of autophagy, collectively termed ‘selective autophagy’, which target specific cellular components. These include mitophagy (removal of damaged mitochondria), xenophagy (clearance of pathogens), ER-phagy (targeting endoplasmic reticulum), lipophagy (breakdown of lipid droplets), and aggrephagy (degradation of protein aggregates). Each selective subtype is regulated by specialized receptor proteins, such as BNIP3, FAM134B, and NDP52, that recognize tagged cargo and mediate their incorporation into autophagosomes via LC3 interaction [26,27,28].

Physiologically, macroautophagy is indispensable for cellular survival, particularly under metabolic stress. It plays key roles in development, immune regulation, tumor suppression, and neural plasticity. Notably, its function in maintaining neuronal health and synaptic integrity is being increasingly appreciated, especially in the context of aging and neurodegeneration. Dysregulation of autophagy has been implicated in a range of pathologies, including neurodegenerative diseases, cancer, inflammatory conditions, and metabolic disorders, underscoring its relevance as both a biomarker and a therapeutic target.

### 2.2. CNS-Specific Features and Physiological Relevance of Autophagy

Autophagy is a critical homeostatic process across all CNS cell types. While neuronal autophagy has traditionally been emphasized due to the high vulnerability of post-mitotic neurons to proteostatic imbalance, increasing evidence shows that autophagy also plays essential roles in glial cells. This section focuses on the physiological relevance of autophagy in maintaining CNS integrity, with a particular emphasis on neurons as prototypical autophagy-dependent cells.

#### 2.2.1. Functional Distinctions from Autophagy in Peripheral Tissues

While basal autophagy is active across various peripheral tissues, CNS autophagy—particularly in long-lived, post-mitotic cells such as neurons and in regulatory glia like astrocytes—plays a more continuous and functionally diverse role, encompassing proteostasis, intercellular communication, synaptic remodeling, and neurovascular integration. Its functions extend beyond catabolic recycling to encompass signal modulation, network remodeling, and long-range organelle transport. Furthermore, the non-renewable nature of neurons, the circuit-level integration of glial cells, and the need for tightly regulated synaptic transmission render CNS autophagy a uniquely tailored process that cannot be fully substituted by the conventional proteasomal or lysosomal degradation pathways typically operating in peripheral tissues.

#### 2.2.2. Neuronal Vulnerability and the Need for Basal Autophagy

Unlike most somatic cells, neurons are post-mitotic, non-dividing cells that must maintain their structural and functional integrity throughout an organism’s lifetime. They cannot dilute damaged proteins or organelles via cell division, rendering them heavily reliant on continuous autophagic flux for intracellular clearance. Indeed, basal (constitutive) autophagy, rather than stress-induced autophagy alone, is essential in neurons for maintaining proteostasis and organelle health. Disruption of core autophagy genes such as ATG5, ATG7, FIP200, and Beclin-1 in neurons results in severe neurodegeneration, marked by axonal swelling, accumulation of ubiquitinated protein aggregates, and progressive neuronal loss, even in the absence of overt external stress [29,30,31,32].

Furthermore, the highly polarized structure of neurons—with elongated axons and intricate dendritic arbors—necessitates long-distance trafficking of autophagosomes. Autophagy is initiated distally in axon terminals and retrogradely transported to the soma for lysosomal degradation [33], a process that integrates motor proteins like dynein with autophagic machinery to ensure spatially precise degradation.

#### 2.2.3. Autophagy in Synaptic Function and Plasticity

Autophagy also plays a pivotal role in synaptic maintenance and remodeling, processes essential for learning and memory. Synapses are sites of intense protein turnover, and autophagy contributes to the regulated degradation of synaptic proteins such as AMPA and GABA receptors [34,35], SNARE components [36,37,38], and neurotransmitter transporters [38]. Evidence indicates that autophagic vacuoles are dynamically recruited to active synaptic zones, where they selectively eliminate dysfunctional components, supporting synaptic pruning, turnover, and long-term potentiation/depression (LTP/LTD) [39,40,41].

In this way, autophagy serves as a local quality control system within neuronal subcompartments, enabling rapid adaptation of synaptic strength in response to activity and environmental changes.

### 2.3. Astrocytic Autophagy as a Central Regulator of Physiological CNS Homeostasis

While research on autophagy in the CNS has traditionally centered on its roles in neuronal survival and synaptic plasticity, it is now clear that non-neuronal cells—especially astrocytes—also utilize autophagic processes to maintain brain health. Astrocytes engage autophagy through mechanisms that are both unique to these glial cells and complementary to neuronal pathways. As key players in metabolic regulation, redox homeostasis, and the maintenance of the extracellular environment, astrocytes depend on autophagic activity not only for their own cellular upkeep but also to safeguard the function and viability of neighboring neural circuits.

This section explores the physiological significance of autophagy within astrocytes, emphasizing its contributions to CNS stability under normal conditions. It highlights both the cell-intrinsic quality control functions and its broader, non-cell-autonomous effects on neural network health.

#### 2.3.1. Energetic Adaptation and Lipid Homeostasis

Astrocytes serve as central hubs for CNS metabolic regulation, notably through their ability to store glycogen and buffer extracellular energy demands [42,43]. Under increased neuronal activity or nutrient deprivation, astrocytic autophagy mobilizes lipid droplets via lipophagy to supply energy substrates, particularly fatty acids for mitochondrial oxidation [44]. Inhibition of autophagy impairs this adaptive metabolic flexibility, leading to mitochondrial dysfunction and reduced ATP production in astrocytes [45]. Additionally, lipid droplet accumulation and lysosomal dysfunction in astrocytes, especially under APOE4 genotype, further underscores the importance of autophagic lipid turnover in maintaining energy homeostasis [46]. Therefore, proper autophagic flux in astrocytes is essential for preventing lipotoxicity, maintaining mitochondrial integrity, and supporting neuronal survival during metabolic challenges [47,48].

#### 2.3.2. Redox Homeostasis and ROS Regulation

Astrocytes play a crucial role in redox balance, particularly in protecting neurons from oxidative stress in metabolically active regions [49,50]. Astrocytes possess a higher rate of mitochondrial ROS production compared to neurons, which is linked to their unique mitochondrial complex I configuration and may play a role in redox signaling and adaptation [51]. While physiological mitophagy in astrocytes remains incompletely characterized, mitochondrial autophagic processes have been linked to modulation of redox signaling [52]. Experimental exposure of astrocytes to carbon monoxide (CO)—a known modulator of redox pathways—has been shown to induce mitochondrial remodeling and antioxidant responses, suggesting a redox-regulatory role potentially involving autophagy signaling [53]. These evidences highlight the multifaceted role of astrocytes in redox homeostasis and ROS regulation, emphasizing the importance of mitochondrial dynamics and autophagic processes in their antioxidant and neuroprotective functions.

#### 2.3.3. Regulation of Neurotransmitter Transport and Membrane Protein Availability

Astrocytes are essential for maintaining neurotransmitter homeostasis in the central nervous system, primarily through high-affinity uptake systems that clear glutamate from the synaptic cleft [54]. The main astrocytic glutamate transporters, GLT-1 (EAAT2) and GLAST (EAAT1), rapidly remove excess glutamate, preventing excitotoxicity and ensuring proper synaptic signaling [55,56]. A recent study identified autophagy as a key regulator of protein trafficking at the astrocytic endfeet, particularly at the neurovascular interface, impacting the polarity and localization of EAAT2 and other membrane transporters [6]. Since disruption of astrocyte glutamate uptake leads to increased extracellular glutamate, impaired synaptic transmission, and increased risk of neurodegeneration [57], these findings suggest that autophagy may contribute to extracellular stability through dynamic control of transporter turnover and membrane composition.

### 2.4. Autophagy Dysfunction in Neurons and Astrocytes in Neurodegenerative Diseases

**Figure 1:**
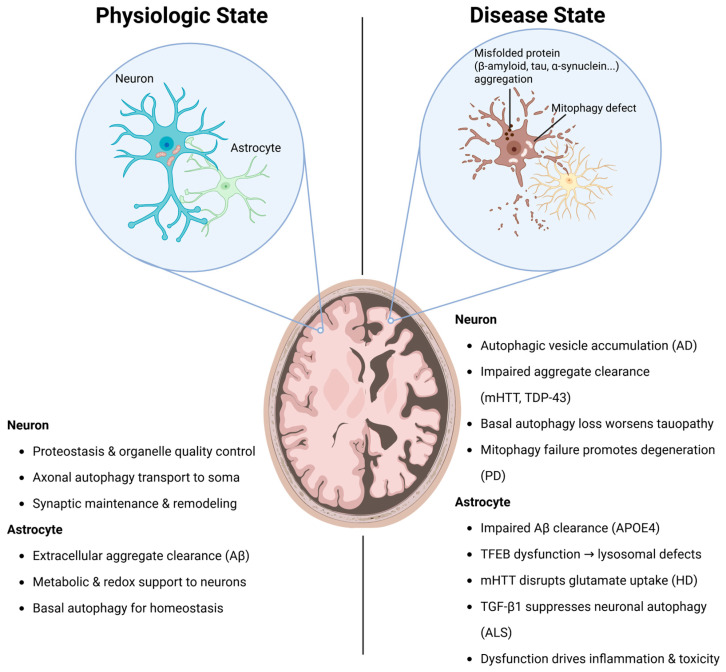
Autophagy in Neurons and Astrocytes: From Physiological Maintenance to Neurodegenerative Dysfunction. Autophagy supports proteostasis and homeostasis in healthy neurons and astrocytes. In disease, autophagy impairment contributes to protein aggregation, neurotoxicity, and inflammatory glial responses. The figure was created in https://BioRender.com.

While autophagy is essential for maintaining cellular integrity under physiological conditions, its dysfunction has been increasingly associated with the development of neurodegenerative diseases. Impaired autophagy leads to the accumulation of aggregated proteins and damaged organelles, triggering cellular stress and neuronal toxicity (Figure 1). In this review, we primarily focus on macroautophagy, the canonical form of autophagy responsible for sequestering cytoplasmic proteins and organelles into double-membrane autophagosomes for lysosomal degradation. Nevertheless, the endosomal–lysosomal pathway, which originates from endocytosis and mediates the turnover of membrane proteins and extracellular cargo, also converges with autophagy at the lysosome. Although distinct in origin and cargo selection, both pathways constitute integral parts of the broader autophagy–lysosomal system. Dysregulation of endosomal trafficking, as observed in mutations of DNAJC6, DNAJC13, or GBA, frequently overlaps with autophagy dysfunction and contributes to impaired proteostasis in neurodegenerative diseases.

Experimental studies have shown that disruption of core autophagy machinery, such as ATG5 or ATG7, is sufficient to induce progressive neurodegeneration in animal models, underscoring the indispensable role of autophagy in neuronal survival [30,31]. Although much of the current research has focused on neurons, recent studies suggest that glial cells—particularly astrocytes—also influence disease progression by modulating autophagic activity, both within themselves and through their interactions with surrounding neural cells [58,59,60].

#### 2.4.1. Alzheimer’s Disease

In Alzheimer’s disease (AD), disruption of autophagic flux results in the accumulation of autophagic vesicles in dystrophic neurites. These vesicles, filled with undigested material, reflect a failure of autophagosome maturation and fusion with lysosomes, which is essential for degrading toxic proteins such as amyloid-β (Aβ) and hyperphosphorylated tau [58,61]. A decline in Beclin-1 expression has been observed in AD brains and is associated with increased Aβ burden and neuronal loss [58,62]. Furthermore, *SQSTM1/p62*, an autophagy adaptor responsible for selective degradation of aggregated proteins, accumulates in AD models, suggesting impaired clearance mechanisms [63].

Astrocytes play a complementary but distinct role in AD pathology. They participate in Aβ clearance by internalizing and degrading extracellular Aβ aggregates. However, this function is compromised in astrocytes expressing the APOE4 allele, the major genetic risk factor for sporadic AD [60,64]. These astrocytes exhibit reduced autophagy, leading to impaired Aβ degradation, a phenotype that can be partially rescued by pharmacological activation of autophagy pathways, including the upregulation of Transcription factor EB (TFEB) [60,65]. Recent findings have further highlighted that TFEB dysregulation impairs lysosomal biogenesis and autophagosome clearance, amplifying Aβ and tau [66]. In addition, age-related loss of neuronal autophagy competency appears to exacerbate tau pathology [67]. Additionally, rare mutations in the endosomal-lysosomal gene CHMP2B—linked to frontotemporal dementia (FTD) but with overlapping AD pathology—disrupt autophagosome maturation, further underscoring the centrality of lysosomal clearance in AD [68].

#### 2.4.2. Parkinson’s Disease

Parkinson’s disease (PD) is characterized by the loss of dopaminergic neurons in substantia nigra pars compacta and the formation of Lewy bodies, which contain aggregates of α-synuclein. Defective autophagy, particularly mitophagy is a key pathological mechanism. Mutations in PINK1 or Parkin, both regulators of mitophagy, impair mitochondrial quality control and increase neuronal susceptibility to degeneration [58,69].

Astrocytes also contribute to PD pathology through autophagy-related mechanisms. While they can internalize extracellular α-synuclein and limit its spread, chronic accumulation may provoke inflammatory responses and promote neurodegenerative propagation. Importantly, PD-associated mutations in LRRK2 (leucine-rich repeat kinase 2) and GBA (encoding the lysosomal enzyme glucocerebrosidase) intimately linked to autophagic and lysosomal pathways—have been shown to impair α-synuclein clearance in astrocytes, leading to intracellular accumulation and enhanced neurotoxicity in co-culture models [60,70]. Moreover, defects in DNAJC6 and DNAJC13, which regulate endosomal trafficking, have been identified in rare familial PD forms and are associated with impaired autophagosome transport and recycling [71]. In addition, LRRK2-related lysosomal dysfunction has been shown to delay autophagosome clearance, and mitochondrial DAMPs (damage-associated molecular patterns) released due to impaired mitophagy may propagate neuroinflammation [72]. These findings support a dual mechanism of α-synuclein toxicity and mitophagy failure in PD.

#### 2.4.3. Huntington’s Disease

Huntington’s disease (HD) is caused by CAG repeat expansions in the huntingtin (HTT) gene, producing a mutant protein (mHTT) that forms aggregates. In HD neurons, although autophagosomes form normally, their ability to recognize and sequester mHTT is impaired, reducing degradation efficiency [58].

In astrocytes, mHTT expression leads to a reduction in the glutamate transporter GLT-1, impairing glutamate uptake and increasing excitotoxic risk. Pharmacological activation of autophagy in these cells—via rapamycin or trehalose—has been shown to restore GLT-1 levels, reduce mHTT burden, and improve astrocytic function, which in turn supports neuronal survival [60,73]. Recent work suggests that mHTT interferes with cargo recognition and endosomal sorting, despite intact autophagosome formation, thus functionally uncoupling initiation from degradation [74].

Emerging evidence also implicates WDR45 (also known as WIPI4), an autophagy regulator, in related disorders such as β-propeller protein-associated neurodegeneration (BPAN). Though genetically distinct from HD, WDR45 dysfunction leads to impaired autophagosome formation and iron accumulation in the brain—pathologies that overlap with aspects of HD-related degeneration [75].

#### 2.4.4. Amyotrophic Lateral Sclerosis

Amyotrophic lateral sclerosis (ALS) involves progressive degeneration of motor neurons, often associated with aggregated proteins such as superoxide dismutase 1 (SOD1) and TAR DNA-binding protein 43 (TDP-43). These aggregates disrupt autophagic pathways and contribute to neuronal toxicity [58,76].

Astrocytes expressing ALS-associated mutations exacerbate disease progression in a non-cell-autonomous manner. Notably, conditioned media from ALS astrocytes can suppress neuronal autophagy through secreted factors like TGF-β1, which has recently been shown to inhibit autophagy via mechanistic target of rapamycin (mTOR) activation in motor neurons [60,77]. These findings suggest that astrocytic autophagy plays a critical regulatory role in maintaining neuronal homeostasis, and its dysfunction may indirectly promote motor neuron degeneration. Additionally, dysregulated stress granule dynamics and impaired clearance of TDP-43 aggregates via selective autophagy have emerged as key contributors to ALS pathology, particularly in the context of persistent stress granules containing TDP-43 [78].

Several autophagy-related genes are also genetically linked to ALS pathogenesis. These include *SQSTM1/p62* and *TBK1*, both of which participate in autophagy receptor signaling and immune regulation [79,80]. Mutations in *TBK1* impair phosphorylation of key autophagy effectors, reducing mitophagic and aggrephagic clearance. *OPTN* (Optineurin), another ALS-linked gene, functions as a cargo receptor in mitophagy; its mutations disrupt mitochondrial quality control and are found in both familial and sporadic ALS cases [81].

## 3. Autophagy in Astrocytes and the Crosstalk with Other Cell Types

Building upon the preceding discussion of CNS-wide autophagic mechanisms and their relevance in neurodegenerative pathology, it is increasingly evident that astrocytes occupy a central position in modulating the functional and pathological landscape of the brain through autophagy.

Astrocytes, as the most abundant glial cells in the CNS [82,83], serve as dynamic regulators of neural and vascular environments, continuously communicating with endothelial cells, microglia, and oligodendrocytes to maintain homeostasis. Recent research has highlighted autophagy as a pivotal mechanism by which astrocytes orchestrate this crosstalk, coordinating responses to metabolic stress, inflammation, and injury.

Rather than functioning in isolation, astrocytic autophagy modulates key aspects of glial communication, including cytokine signaling, oxidative balance, and clearance of cellular debris—making it an integral component of intercellular homeostasis and neuroprotection in both physiological and pathological contexts. In this chapter, we examine how autophagy within astrocytes contributes to functional interactions with neighboring cell types, and how disruptions in this regulatory axis may underlie a range of neurological disorders.

### 3.1. Astrocyte-Neuronal Crosstalk

Astrocytes and neurons maintain a bidirectional relationship that is fundamental for CNS homeostasis. Autophagy in astrocytes is increasingly recognized as a crucial regulator of this crosstalk, influencing synaptic activity, neurotransmitter turnover, and neuronal survival. By modulating glutamate clearance, metabolic support, and release of gliotransmitters, astrocytic autophagy contributes to both protective and pathological outcomes in neural circuits [84,85,86]. Astrocytes exhibit robust basal autophagic activity, which safeguards neuronal function by regulating neurotransmitter turnover, providing metabolic support, and maintaining ion equilibrium [4,6]. Emerging evidence indicates that astrocytic autophagy promotes neuronal survival through multiple mechanisms, including the clearance of pathogenic proteins such as amyloid-β and α-synuclein, the modulation of microglial activity, and the release of inflammatory mediators [87,88].

Recent studies have uncovered a novel intercellular pathway in which neuronal autophagosomes are transferred to astrocytes for degradation. In both human and murine models, neuronal autophagosomal vesicles are released under conditions of suppressed synaptic activity and subsequently internalized by astrocytes, where they undergo lysosomal fusion and degradation. This process relies on dynamin- and cholesterol-dependent endocytosis, occurs independently of direct cell–cell contact, and enables astrocytes to recycle neuron-derived materials efficiently [89]. Such neuron-to-astrocyte autophagy transfer provides a more rapid route for autophagosome clearance than conventional axonal retrograde transport and has been proposed as a potential regulatory mechanism in maintaining brain homeostasis and mitigating neurodegenerative pathology. Another study demonstrated that neurons expressing pathogenic LRRK2 upregulate secretory autophagy and the compensatory release of exosomes to mediate waste disposal and transcellular communication, respectively [90]. Consequently, targeting astrocytic autophagy has been proposed as a potential therapeutic strategy for neurodegenerative diseases including AD and PD.

Together, these findings establish astrocytic autophagy as a key regulator of neuron–astrocyte communication. By coordinating neurotransmitter clearance, metabolic support, and synaptic signaling, it directly shapes neuronal function. Its disruption drives excitotoxicity, synaptic impairment, and neurodegeneration, positioning astrocytic autophagy as a compelling therapeutic target for maintaining neuronal integrity.

### 3.2. Astrocyte-Endothelial Crosstalk

Astrocytes and vascular endothelial cells interact closely to maintain CNS homeostasis, with autophagy playing a key regulatory role in this crosstalk by modulating cellular energy balance [91,92]. In astrocytes, autophagy influences neuroinflammatory responses and facilitates adaptation to pathological stress. While direct evidence of astrocytic autophagy affecting endothelial dysfunction in neurodegeneration is limited, emerging studies suggest that maintaining astrocytic autophagy may be critical to preserve blood–brain barrier (BBB) integrity and neurovascular unit (NVU) function.

Astrocytes support BBB integrity by releasing growth factors, morphogens, and extracellular vesicles that regulate endothelial tight junction proteins [93]. Although direct evidence linking autophagy to astrocyte–endothelial crosstalk in neurodegeneration is limited, emerging studies suggest indirect mechanisms. Astrocytes influence endothelial function through cytokine secretion and metabolic signaling [94,95], and co-culture models show astrocytic modulation of endothelial cytokine profiles under both homeostatic and inflammatory conditions [96]. Autophagy likely contributes by regulating inflammatory cytokine release, ROS signaling, and mitochondrial function [97]. Reactive astrocytes secrete factors such as vascular endothelial growth factor (VEGF) and interleukin-6 (IL-6), which compromise endothelial tight junctions. In viral encephalitis models, astrocyte-derived VEGF and IL-6 degrade zonula occludens-1 (ZO-1) and claudin-5 via proteasomal and autophagy-associated pathways [98]. Additionally, astrocytic HMGB1 (High Mobility Group Box 1), a DAMP molecule, promotes endothelial progenitor cell-mediated vascular remodeling, linking astrocytic stress responses to vascular repair in stroke, a pathology often accompanied by neurodegeneration-associated vascular dysfunction [99].

### 3.3. Astrocyte-Microglial Crosstalk

Astrocytes play a central role in neuroinflammation, with autophagy emerging as a key regulator of their interaction with microglia. Beyond maintaining cellular homeostasis, astrocytic autophagy modulates inflammatory signaling, cytokine release, and debris clearance—all of which influence microglial activation [100]. RUBICON-dependent noncanonical autophagy enables astrocytes to degrade microglial fragments; disruption of this pathway leads to debris accumulation and heightened microglia-driven inflammation [101]. Autophagy also regulates the secretion of immunomodulatory factors such as chemokine (C-C motif) ligand 7 (CCL7), which increases after traumatic brain injury and activates microglia. Its knockdown reduces inflammatory responses, implicating autophagy in its regulation [102]. Astrocyte–microglia interactions are bidirectional within both physiological and neurodegenerative microenvironments [103,104,105]. Cytokine signaling and debris exchange between these glia are influenced by autophagic activity [106]. In neurotoxic injury models, astrocyte-derived signals—potentially linked to autophagy—induce nitric oxide release and morphological activation in microglia [107,108,109]. Conversely, microglia-derived M1 cytokines can induce reactive astrocytes and impair astrocytic autophagy, contributing to reduced neuroprotection and glutamate clearance under pathological conditions [110].

In the context of AD, microglia play an important role during pathology initiation and progression [111,112] are suggested to prevent premature AD-related lethality [113]. When microglia approach Aβ plaques via chemotaxis, they phagocytose and degrade Aβ within lysosomal compartments through autophagy [114] in a process called MAP1LC3B/LC3-associated phagocytosis [115]. However, long-term exposure to Aβ disrupts microglia’s ability to degrade it through autophagy [116]. Also, there is a synergistic interplay of astrocytes and microglia in transfer processing of Aβ aggregates from astrocytes to microglia, which may be an important mechanism for the clearance of protein aggregates [117]. For instance, central complement factor C3 secreted from astrocytes interacts with microglial C3a receptor (C3aR) to mediate Aβ pathology and neuroinflammation in AD mouse models [118].

In PD, astrocytes and microglia can mutually modulate each other’s activity and function as collaborative contributors to the exacerbation of dopaminergic neuron degeneration [119]. Both astrocytes and microglial activation and iron dyshomeostasis are crucial events in its pathogenesis [120]. For example, blocking A1 astrocyte conversion by microglia is neuroprotective, it could prolong life and reduce neuropathological and behavioral deficits in a human A53T mutant α-syn transgenic mouse model [121]. Inflammatory cytokines produced by active microglia and astrocytes also could upregulate divalent metal transporter 1 (DMT1) and downregulate ferroportin1 (FPN1), resulting in iron accumulation in neurons [122].

In ALS, the shift in astrocytes from a neuroprotective to a neurotoxic state has been shown to coincide with alterations in microglial phenotype, indicating that astrocytes may play a key role in modulating microglial activation and neuroinflammatory responses [123,124]. This shift is closely linked to dynamic interactions with other glial cells, particularly microglia. Multiple studies have demonstrated that pathological microglia contribute to shaping astrocyte phenotypes that promote disease progression in ALS [125,126]. Microglia are activated earlier than astrocytes in response to cellular stress or injury, initiating NF-κB signaling and releasing pro-inflammatory cytokines such as TNF-α and IL-1β [127]. These cytokines impair the function of connexin-43 (Cx-43), the primary gap junction protein in astrocytes, thereby compromising their neuroprotective capacity [128].

Taken together, these findings underscore the role of astrocytic autophagy as a crucial mediator in glial crosstalk. By influencing debris processing, chemokine secretion, and stress-induced signaling, autophagy enables astrocytes to actively shape microglial behavior and inflammatory tone in the CNS. Disruption of this pathway may thus contribute to persistent microglial activation and chronic neuroinflammation in a variety of neurological diseases, providing a promising target for future therapeutic strategies.

### 3.4. Astrocyte-Oligodendrocyte Crosstalk

Astrocyte-oligodendrocyte crosstalk plays a pivotal role in maintaining myelin integrity and CNS homeostasis, with recent studies highlighting the importance of both signaling and autophagy-related mechanisms in this glial interplay [129,130]. Although not astrocyte-specific, glial autophagy appears to be essential for clearing aggregated myelin proteins and maintaining sheath integrity [131]. Dysregulation of this process disrupts myelin compaction and causes structural damage, highlighting the supportive role of glia, although the precise mechanisms remain to be fully elucidated [132]. Notably, astrocyte-derived signaling molecules, such as ephrin-B1 are critical for oligodendrocyte development and myelination; deletion of astrocytic ephrin-B1 results in reduced oligodendrocyte numbers and impaired myelin formation, indicating that astrocyte signaling modulates oligodendrocyte lineage progression [133].

At the molecular level, the Nrf2 (nuclear factor erythroid 2–related factor 2)-cholesterol axis has emerged as a key regulator of astrocyte-oligodendrocyte communication. Sustained activation of Nrf2 in astrocytes suppresses cholesterol biosynthesis, thereby inhibiting remyelination, while inhibition of Nrf2 restores cholesterol efflux and supports oligodendrocyte function [130,134]. Cholesterol derivatives supplied by astrocytes are indispensable for oligodendrocyte membrane synthesis and myelin repair, further emphasizing the metabolic interdependence between these glial cells [135]. Additionally, astrocyte-oligodendrocyte gap junctions, composed of connexins, such as Cx43 and Cx30, facilitate the exchange of ions and metabolites, and their disruption leads to myelin decompaction and axonal dysfunction [6,136].

Glutamate homeostasis is another critical aspect of astrocyte-oligodendrocyte crosstalk. Astrocyte ablation results in elevated extracellular glutamate levels, which can trigger N-Methyl-D-aspartic acid (NMDA) receptor-mediated oligodendrocyte injury and myelin decompaction [132,137]. Pharmacological blockade of NMDA receptors has been shown to preserve oligodendrocyte integrity in the absence of astrocytic support, highlighting the neuroprotective role of astrocytes in buffering excitotoxic insults [138].

**Figure 2:**
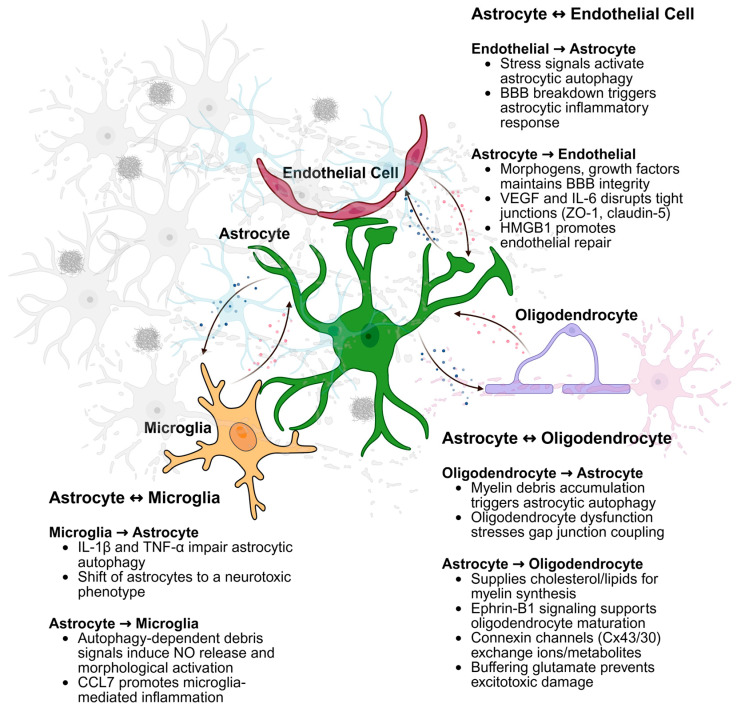
Astrocytic Autophagy-Mediated Crosstalk with CNS Cell Types. Astrocytic autophagy regulates bidirectional communication with endothelial cells, microglia, and oligodendrocytes, coordinating stress responses, inflammation, and metabolic support essential for CNS homeostasis. The figure was created in https://BioRender.com.

Collectively, these findings underscore the complex molecular networks that govern astrocyte-oligodendrocyte interactions in the CNS (Figure 2). Disruption of these networks—whether through impaired signaling, metabolic dysfunction, or loss of autophagic homeostasis—may contribute to the pathogenesis of demyelinating and neurodegenerative diseases, and targeting these pathways holds promise for novel therapeutic interventions.

## 4. Astrocytic Autophagy-Targeted Drug Development Progress

Given the significant involvement of astrocytes in neurological disorders and the critical role of autophagy in maintaining cellular health, targeting astrocytic autophagy has emerged as a promising therapeutic strategy. The therapeutic potential of modulating autophagy in astrocytes arises from its multifaceted involvement in the pathogenesis of neurological diseases, including the clearance of toxic protein aggregates, maintenance of cellular homeostasis, and support of neuronal function [60].

In the following sections, we will examine pharmacological agents that modulate astrocytic autophagy, with a particular focus on their regulatory mechanisms in neurodegenerative diseases. In the final section, we will review the most recent clinical trial outcomes related to autophagy modulation in the context of neurodegenerative disorders. However, definitive clinical evidence demonstrating the direct effects of autophagy-modulating agents on astrocytes remains limited, as many studies rely on broad cellular effects.

### 4.1. Pharmacological Agents Targeting Astrocytic Autophagy

Preclinical studies have demonstrated that pharmacological agents such as rapamycin, lithium, and certain antidepressants yield encouraging results in models of ischemic stroke, AD, PD, HD, ALS, lysosomal storage disorders, and mood disorders [60,139,140] (Table 1). However, translating these findings into effective clinical treatments remains challenging. Key obstacles include the complex and sometimes dualistic role of autophagy in disease, the difficulty of achieving cell type-specific drug delivery, and the need for a deeper understanding of the long-term consequences of modulating autophagy [141,142,143].

This section aims to examine pharmacological efforts to modulate autophagy in astrocytes as a potential therapeutic approach for neurological diseases. Specifically, it will review the pharmacological agents employed, the target diseases, the mechanisms of action, the reported outcomes, and the potential challenges associated with this therapeutic strategy.

Regulation of autophagy in astrocytes can be achieved through a broad range of compounds classified as either inducers or inhibitors of autophagy [144]. These agents exert their effects via diverse mechanisms, including mTOR inhibition, AMPK activation, modulation of Beclin-1, and inhibition of lysosomal function [144].

Rapamycin, a well-known immunosuppressant, serves as a potent autophagy inducer in astrocytes by inhibiting the mTOR signaling pathway [60]. Lithium, widely used in the treatment of mood disorders, has also been shown to induce autophagy in astrocytes by inhibiting inositol monophosphatase [139]. Certain antidepressants, such as amitriptyline and citalopram, have been found to influence autophagy pathways in astrocytes, potentially via mechanisms involving acid sphingomyelinase (ASM) inhibition and modulation of PI3 kinase-dependent pathways [145]. Dexmedetomidine, an α2-adrenergic receptor agonist, has demonstrated the ability to inhibit neuronal autophagy in the context of ischemia–reperfusion injury [146]; however, its effects on astrocytic autophagy may vary depending on the specific pathological context.

Ginkgolide K, a component of Ginkgo biloba extract, may induce protective autophagy in astrocytes following oxygen-glucose deprivation by activating the AMPK/mTOR/ULK1 signaling pathway [147]. A novel indole alkaloid derivative, IADB, has been shown to enhance autophagy and facilitate the clearance of protein aggregates in motor neuron-like cells, suggesting its potential relevance in astrocytes [148]. Bafilomycin A1, a well-established autophagy inhibitor, is frequently used in studies investigating autophagic flux in astrocytes [142]. In addition, caloric restriction mimetics such as resveratrol and metformin have been reported to modulate autophagy in astrocytes [60]. Beclin-1, a key protein in the autophagic pathway, has also emerged as a notable pharmacological target in astrocytes [149].

The fact that many of these autophagy-regulating agents were originally developed for other therapeutic purposes—such as immunosuppression, mood stabilization, and treatment of mood disorders—underscores the potential of drug repurposing in targeting astrocytic autophagy for neurological disease therapy. Leveraging already approved drugs with established safety profiles may accelerate the development of novel treatments [150].

Autophagy exerts a paradoxical influence in neurodegenerative diseases, functioning both as a protective mechanism that clears toxic protein aggregates and damaged organelles, and as a potential cytotoxic pathway when excessively or aberrantly activated [144]. This dual role poses a major challenge for therapeutic development, as global enhancement of autophagy may promote neuroprotection in early disease stages but could accelerate neuronal loss if not precisely regulated with respect to timing, dose, and cellular context. Recent findings highlight that the therapeutic efficacy of autophagy modulation depends critically on the disease stage or the specific cellular environment, underscoring the necessity of strategies that allow selective fine-tuning of autophagic flux rather than indiscriminate up- or down-regulation [87,151,152]. Identifying reliable biomarkers to monitor autophagic activity will be essential for guiding individualized and context-dependent therapeutic approaches [153].

### 4.2. Regulation of Astrocytic Autophagy in Specific Neurological Disorders

#### 4.2.1. Alzheimer’s Disease

Studies related to astrocytic autophagy in Alzheimer’s disease have reported that astrocyte-specific overexpression of key autophagy-related proteins such as LC3B in AD mouse models leads to a reduction in Aβ aggregates and improvement in cognitive function [154]. Pharmacological agents that enhance autophagy, including rapamycin, metformin, and resveratrol, have been shown to enhance the Aβ clearance capacity of astrocytes in preclinical models [60]. However, further research is ongoing to determine the optimal strategies for targeting this pathway [155].

#### 4.2.2. Parkinson’s Disease

In Parkinson’s disease, studies have investigated the induction of astrocytic autophagy by lithium and its protective effects against MPP+-induced toxicity [139]. Other autophagy-modulating agents, such as rapamycin, trehalose, and valproate, have demonstrated neuroprotective effects in animal models; however, studies specifically focusing on astrocytic autophagy remain relatively limited [156].

#### 4.2.3. Amyotrophic Lateral Sclerosis

In amyotrophic lateral sclerosis (ALS), the potential of IADB to regulate autophagy has been explored in preclinical studies, demonstrating reductions in mutant SOD1 aggregates and attenuation of astrocyte activation [148]. Autophagy plays a complex role in ALS, and various autophagy-targeting compounds are currently under investigation. Given the limited success of existing ALS therapies, targeting astrocytic autophagy remains an active and promising area of research [157].

### 4.3. Autophagy-Modulating Drugs in Clinical Development for Neurodegenerative Diseases

#### 4.3.1. Blarcamesine (ANAVEX^®^2-73)—Alzheimer’s Disease

Blarcamesine (ANAVEX^®^2-73) is a novel, orally available small molecule that acts as an activator of the Sigma-1 receptor (SIGMAR1) [158] (Table 2). By activating SIGMAR1, Blarcamesine induces autophagy, a critical pathway for the removal of protein aggregates and misfolded proteins [140,158]. In Alzheimer’s disease, autophagy impairment is known to precede the formation of Aβ and tau aggregates [159], and Blarcamesine has the potential to interrupt this progression in its early stages. Beyond autophagy, SIGMAR1 activation by Blarcamesine is also associated with glutamate regulation, maintenance of endoplasmic reticulum function, calcium homeostasis, neurogenesis promotion, reduction in ROS, suppression of neuroinflammation, and attenuation of Aβ toxicity [160,161]. This multifaceted and upstream action may overcome the limitations of traditional therapies that target a single pathological mechanism, potentially providing more sustained clinical benefits by restoring cellular homeostasis at a fundamental level.

Blarcamesine has successfully completed Phase 2a and Phase 2b/3 clinical trials for AD [162]. The pivotal Phase 2b/3 trial (ANAVEX2-73-AD-004, NCT03790709) was a 48-week, multicenter, randomized, double-blind, placebo-controlled study involving 508 patients with mild cognitive impairment or mild dementia across 52 medical research centers in five countries. At 48 weeks, clinical decline was reduced by 36.3% overall on the ADAS-Cog13 scale, with reductions of 34.6% in the 30 mg group and 38.5% in the 50 mg group [162].

Blarcamesine showed a favorable safety profile with no neuroimaging-related adverse events. The most common adverse event was dizziness, generally transient and of mild to moderate severity [162].

#### 4.3.2. Rapamycin (Sirolimus)—Alzheimer’s Disease

Rapamycin is currently being actively investigated in clinical trials for Alzheimer’s disease [163]. An initial open-label Phase 1 pilot study evaluated CSF drug concentrations and safety in patients with mild cognitive impairment (MCI) or early AD [163]. However, rapamycin was not detected in the CSF either before or after treatment [164], raising concerns about whether therapeutic concentrations can be achieved in the brain, despite some changes in biomarkers.

#### 4.3.3. Felodipine—Huntington’s Disease

Felodipine is an L-type calcium channel blocker approved for the treatment of hypertension [165]. It was identified through drug repurposing screens as an autophagy-inducing agent in preclinical models [166]. Importantly, preclinical pharmacokinetic studies showed that felodipine achieved good brain penetrance at plasma concentrations comparable to those in humans [166].

Felodipine is currently being evaluated in the FELL-HD trial (Felodipine for Early-stage HD, ISRCTN56240656), a Phase 2, single-center, open-label, dose-finding study [167]. The trial aims to enroll 18 participants, who will receive felodipine for 58 weeks, followed by a 4-week follow-up period. Participants are randomly assigned to one of three dosage groups (5 mg, 10 mg, or 20 mg daily), with dose escalation based on tolerability. The primary objective is to assess safety and tolerability based on the number of drug-related adverse events. Secondary outcomes include assessments of motor and cognitive function, non-motor symptoms, quality of life, and biomarker evaluations through brain MRI and blood/CSF analyses [167]. Drug repurposing strategies such as that of felodipine offer significant advantages by leveraging the known safety, affordability, and pharmacological profiles of FDA-approved drugs, allowing for accelerated clinical development by bypassing early-phase safety and pharmacokinetic studies.

#### 4.3.4. AT-1501 (Tegoprubart)—Amyotrophic Lateral Sclerosis (ALS)

AT-1501 (Tegoprubart) is a humanized monoclonal antibody designed to antagonize the immune signaling molecule CD40L [168]. The CD40L pathway is overactivated in patients with motor neuron diseases (MND), including ALS [169].

By blocking CD40L, AT-1501 aims to inhibit immune-mediated inflammatory responses, thereby slowing disease progression [168]. Although AT-1501 does not directly target autophagy, CD40 is expressed on CNS immune cells such as microglia [170], and the interplay between autophagy and inflammation suggests that its anti-inflammatory effects could indirectly support autophagic function. Thus, even without directly modulating autophagy, improving the inflammatory environment may facilitate proteostasis and cellular health [171].

AT-1501 has completed a Phase 2a, multicenter, multiple-dose study (NCT04322149) involving 54 adult ALS patients [172]. Topline results indicated that Tegoprubart was safe and well tolerated, achieved dose-dependent target engagement, and reduced inflammatory biomarkers associated with ALS [172].

**Table 1:** Effects of autophagy-modulating agents in neurodegenerative diseases.

Neurological Disorder	Pharmacological Agent	Mechanism of Modulation in Astrocytes (If Known)	Key Preclinical Outcomes
Alzheimer’s Disease	LC3B Overexpression (Genetic)	Enhanced autophagy	Reduced Aβ aggregates, improved cognition in mouse models [155]
Alzheimer’s Disease	Rapamycin	mTOR inhibition	Promotes Aβ clearance [173]
Alzheimer’s Disease	Resveratrol, Metformin	Caloric restriction mimetics	Modulates autophagy, potential Aβ clearance [174]
Parkinson’s Disease	Lithium	Inhibition of inositol monophosphatase	Protective effects against MPP+-induced injury [139]
Huntington’s Disease	Rapamycin	mTOR inhibition	Reduced mHTT accumulation [175]
ALS	IADB	Autophagy promotion	Reduced mutant SOD1 aggregates, alleviated astrocyte activation in mouse models [148]
Mood Disorders	Amitriptyline, Citalopram	PI3 kinase-dependent pathways, ASM inhibition (potential)	Induction of autophagy in astrocytes [145]

**Table 2:** Autophagy-Modulating Drugs in Clinical Trials for Neurodegenerative Diseases.

Drug Name	Primary Neurodegenerative Disease(s)	Primary Autophagy-Related Mechanism	Current/Latest Clinical Trial Phase	Relevant ClinicalTrials.gov/ISRCTN ID(s)	Key Clinical Outcome (Brief)
Blarcamesine (ANAVEX^®^2-73)	AD, PD	SIGMAR1 activation (autophagy enhancement)	AD: Phase 2b/3 completed; PD: Phase 2 PoC completed	NCT03790709, NCT04314934	AD: Significantly slowed clinical progression (ADAS-Cog13, CDR-SB), improved biomarkers (plasma Aβ42/40-ratio, brain volume)
Rapamycin (Sirolimus)	AD	mTOR inhibition (autophagy induction)	AD: Phase 2 recruiting (NCT04629495), Phase 1 completed (NCT04200911)	NCT04629495, NCT04200911 1	AD: Phase 1 showed rapamycin not detectable in CSF, but changes in AD/inflammatory biomarkers; Phase 2 ongoing for safety, tolerability, feasibility
Felodipine	HD	L-type Calcium Channel Blocker (autophagy induction)	HD: Phase 2 (dose-finding)	ISRCTN56240656, EudraCT-2021-000897-27	HD: Primary outcome is safety and tolerability; exploratory outcomes include motor/cognitive function, biomarkers
AT-1501 (Tegoprubart)	ALS	CD40L antagonism (indirect link via inflammation/immune modulation)	ALS: Phase 2a completed	NCT04322149	ALS: Safe and well-tolerated, demonstrated dose-dependent target engagement, reduced inflammatory biomarkers

## 5. Conclusions

Astrocytic autophagy plays a pivotal role in maintaining CNS homeostasis by regulating synaptic function, neuroinflammation, and intercellular communication with other glial and neuronal cells. Dysregulation of this process contributes to the pathogenesis of various neurodegenerative diseases by promoting protein aggregation, chronic inflammation, and disruption of the neurovascular unit. Accumulating evidence highlights astrocytic autophagy as an integrative mechanism in neuroglial interactions rather than a mere cellular clearance pathway. While pharmacological modulation offers therapeutic potential, challenges such as cell-type specificity and the dual roles of autophagy necessitate further investigation. Advancing our understanding of astrocyte-specific autophagy functions may open new avenues for targeted interventions in neurodegenerative disorders.

## Data Availability

Data are contained within the article.
